# Hepatocyte-like Cells Derived from Human Pluripotent Stem Cells Can Be Enriched by a Combination of Mitochondrial Content and Activated Leukocyte Cell Adhesion Molecule

**DOI:** 10.31662/jmaj.2018-0042

**Published:** 2019-08-06

**Authors:** Hiromi Yamashita, Keiichi Fukuda, Fumiyuki Hattori

**Affiliations:** 1 Department of Cardiology, Keio University School of Medicine, Tokyo, Japan; 2 iPS Stem cell Regenerative Medicine, Kansai Medical University School of Medicine, Hirakata, Japan

**Keywords:** human induced pluripotent stem cell, hepatocyte-like cells, purification method, regenerative cell therapy, pharmaceutical development

## Abstract

**Introduction:**

Non-genetic purification methods for pluripotent stem cell-derived hepatocyte-like cells are useful for liver regenerative therapy and pharmaceutical applications.

**Methods:**

Fluorescent activated cell sorting (FACS) was used to separate cells by combining two parameters: cellular mitochondrial content evaluated by the mitochondrial membrane potential-dependent fluorescent probe (TMRM) and immunocytochemical detection of activated leukocyte cell adhesion molecule (ALCAM). This method was applied to murine fetal, human embryonic stem cell (ESC)-derived, and human induced pluripotent stem cell (iPSC)-derived cell-mixtures. Separately sorted cell fractions were evaluated by quantitative PCR, immunohistochemistry, and cytochemistry for HNF4a, AFP, and albumin mRNA and/or protein expression.

**Results:**

Hepatocyte-like cells were segregated into the high TMRM signal and ALCAM-positive population. The purity of hepatocyte-like cells derived from human iPSCs was 97 ± 0.38% (n = 5).

**Conclusions:**

This hepatocyte-like cell purification method may be applicable to the quality control of cells for liver regenerative cell therapy and pharmaceutical development.

## Introduction

The liver, a highly multifunctional organ, detoxifies various metabolites, synthesizes circulating proteins, and produces bile. Therefore, replacement by an artificial organ is extremely unlikely. Furthermore, in addition to being the largest organ in the human body, the liver can also regenerate itself. However, certain conditions may cause irreversible liver failure, requiring liver transplantation because of compromised regenerative capacity ^(1)^. While the liver is the most frequently transplanted organ in the world, the number of donors is insufficient, and a satisfactory increase in the donor number cannot be expected. Thus, in order to expand the therapeutic potential of liver disease, regenerative cell therapy using human pluripotent stem cells is being investigated as a potential future treatment option.

Numerous studies have described efficient methods for differentiating human pluripotent stem cells into hepatic cells ^(2), (3), (4), (5)^. However, a method which only produces hepatocytes is not currently available. Transplantation of residual stem cells and/or unwanted growth-capable cells may result in tumor formation ^(6)^. For safe therapeutic applications, elimination of these cells is crucial. Purification of hepatocytes is the simplest and most straightforward strategy to achieve this. Also, in pharmaceutical applications, quality-controlled human iPSC-derived hepatocytes would be ideal to predict the metabolism and hepatotoxicity of drug compounds, in combination with general cell-lines and animals.

Previous studies have described cell-surface markers characteristic of several developmental stages and/or cell-types in the hepatic lineage. For multipotent hepatocyte progenitor cells, the cell surface markers CD133 ^(7)^, CD13 ^(8)^, EpCAM ^(9), (10)^, and c-MET ^(11)^ have been identified. Currently, only asialoglycoprotein receptor 1 (ASGR1) is known as a definitive hepatocyte-like cell receptor ^(12)^. In this study, immunological staining for ASGR1 and fluorescence-activated cell sorting (FACS) were utilized to segregate approximately 30% of albumin-positive hepatocyte-like cells derived from human iPSCs.

Activated leukocyte cell adhesion molecule (ALCAM) is known as a marker of fetal mouse ^(13)^ and human iPSC-derived ^(14)^ hepatic stellate cells which do not express albumin or other hepatocyte-related characteristics. In the present study, we found that ALCAM is expressed in human pluripotent stem cell-derived hepatocyte-like cells which are immunologically positive for albumin.

To perform multiple physiological functions, hepatocytes require a substantial amount of ATP, which is primarily produced by mitochondrial oxidative phosphorylation ^(15)^. Accordingly, adult liver hepatocytes possess numerous mitochondria. We previously developed a cardiomyocyte purification method based on cellular mitochondrial contents, where we utilized selected fluorescent probes that accumulate in the mitochondrial matrix driven by membrane potential ^(16)^.

In this study, we focused on the importance of mitochondrial content in reflecting definitive differentiation of hepatocyte-like cells and successfully developed a unique purification method for human pluripotent stem cell-derived hepatocyte-like cells by combining mitochondrial content and ALCAM expression.

## Materials and Methods

### Maintenance of human pluripotent stem cells

Human ESCs (khES-2 strain obtained from Kyoto University) were maintained in an undifferentiated state as described previously ^(16)^. Briefly, human ESCs were co-cultured with mouse embryonic fibroblasts (MEFs) in human ESC medium composed of 80% D-MEM/Ham’s F-12 medium (1/1 ratio; Wako Pure Chemical Industries, Osaka, Japan), 20% Knockout Serum Replacement (KSR; Invitrogen, Carlsbad, CA, USA), 0.1 mM nonessential amino acids (Sigma-Aldrich, St. Louis, MO, USA), 2 mM L-glutamine (Sigma-Aldrich), 0.1 mM β-mercaptoethanol (Sigma-Aldrich), and 4 ng/mL basic fibroblast growth factor (Wako).

Human iPSCs (253G1 strain obtained from Center for iPSC Research and Application, Kyoto University) were maintained under feeder-free conditions in Essential 8^®^ medium (Invitrogen) on culture dishes coated with 0.5 μg/cm^2^ iMatrix-511 (Nippi, Tokyo, Japan).

### Embryoid based-spontaneous differentiation of human ESC

To initiate differentiation, semi-confluent human ESCs were washed thrice and cultured for 1 week with RPMI-1640 medium (Wako). Cell clumps differentiated from human ESCs were collected via 0.1% collagenase (Wako) treatment, and their embryoid bodies (EBs) were formed by floating the culture in ultra-low attachment culture dishes (Corning, Inc., NY, USA) in RPMI-1640:MEMα = 1:1 mixed medium containing 5% fetal bovine serum (Biowest, Nuaillé, France) and 0.5 mM ascorbic acid (Sigma-Aldrich). To obtain mature hepatocyte-like cells, the cells were cultured for approximately 2 months while exchanging the medium every week.

### Directed hepatocyte-like cell differentiation of iPSC

Hepatic differentiation from human iPSCs was performed as described previously ^(5)^ with some modifications. Confluent human iPSCs were passaged at a split ratio of 1:3 on iMatrix-511 with Essential 8^®^ medium and cultured for 2 d. To initiate differentiation, the cells were subsequently treated with RPMI-1640 plus 2% B27 Minus Insulin (RPMI-B27), containing 3-6 μM CHIR-99021 (MCE, NJ, USA #HY-10182) and 1% GlutaMAX^®^ (Invitrogen) for 24 h, followed by treatment with RPMI-B27 alone for 24 h. To initiate differentiation into hepatic progenitors, the cells were treated with RPMI-B27 containing 1% GlutaMAX^®^, and 1% dimethyl sulfoxide (DMSO; Wako) for 5 d. The cells were detached using TrypLE Select (Invitrogen) and re-seeded on culture dishes coated with Matrigel (Growth Factor Reduced; Corning, Inc.) in hepatocyte maturation medium: Leibovitz’s L-15 medium (Wako) containing 8.3% tryptose phosphate broth, 10 μM hydrocortisone 21-hemisuccinate, 50 μg/mL sodium-L-ascorbate, 100 nM dexamethasone (DEX) (all from Sigma-Aldrich), 0.58% insulin-transferrin-selenium (ITS), 2 mM GlutaMAX^®^ (all from Invitrogen), 8.3% fetal bovine serum (Biowest), and 100 nM Dihexa (TRC, Ontario, Canada #H293745). This culture was continued for 20 d to obtain definitively differentiated hepatocyte-like cells. During the culture, the medium was changed every 2 d.

### Experimental animals

Pregnant ICR mice were purchased from Japan CLEA. The experimental procedure and the protocol were approved by the Animal Care and Use Committees of Keio University, Japan. We carried out abdominal surgery on the pregnant ICR mice on post-coital day 14.5 under deep anesthesia. We obtained the embryos and isolated their whole viscera.

### Cell preparation for purification of fetal mouse hepatoblasts

Fetal mice viscera (embryonic day 14.5) were dispersed in single cells as described previously ^(16)^. Shortly, isolated whole viscera was treated with 0.1% collagenase (Wako), 0.083% trypsin (Difco, Detroit, MI, USA), 20 μg ml−1 DNase I (Sigma) in Ads buffer (116 mM NaCl, 20 mM HEPES, 12.5 mM NaH_2_PO_4_, 5.6 mM glucose, 5.4 mM KCl, and 0.8 mM MgSO_4_; pH 7.35) by stirring at 37℃ for 1–5 h. Dispersed cells were applied to FACS experiments.

### Cell preparation for purification of human PSCs-derived hepatocyte-like cells

Human ESC-derived EBs were dispersed into single cells using the same method used for fetal mouse hepatoblast purification. Cells obtained from directed hepatocyte differentiation, were detached from the dishes with 0.1% collagenase (Wako), 0.083% trypsin (Difco, Detroit, MI, USA), 10 μM ROCK inhibitor (Y-27632; Wako), 20 nM cyclosporine A (Wako), and 50 μg/mL sodium-L-ascorbate in Ads buffer by horizontal rotation (approximately 200 rpm) at 37℃ for 2 h. Detached cells were dispersed into single cells by gentle pipetting.

### FACS analyses and purification

All single cells were stained for mitochondria with 100 nM TMRM (tetramethylrhodamine, methyl ester; Invitrogen) in hepatic maturation medium for 30 min at 37℃. Next, the cells were stained with an anti-ALCAM antibody (1:50, R&D Systems, Minneapolis, MN, USA) for 50 min, and subsequently, Alexa Fluor 488 donkey anti-goat IgG (1:100, Invitrogen) for 30 min. These processes were performed on ice using cooled Ads buffer containing 2% fetal bovine serum for washing and diluting the antibodies. The stained cells were analyzed and sorted by FACS Aria™ III (BD Biosciences, Franklin Lakes, NJ, USA) using an 85 or 100 μm nodule. As the common gating strategy to eliminate doublets, the major FSC-A and SSC-A population was gated by FSC-H and -W, followed by gating by SSC-H and -W. For data acquisition and sorting, these single cell-containing droplets were separated by AlexaFlour 488 (FITC-channel) and PE-channel. Flow-rate was optimized for higher sorting efficiency (approximately > 90%). Sorted cells were cultured on Matrigel or MEFs in hepatocyte maturation medium supplemented with 10 μM Y-27632, 20 nM cyclosporine A, with penicillin, streptomycin, and amphotericin (Invitrogen) for approximately 5 d.

### Microarray analysis

Complementary DNA and Amino Allyl aRNA was synthesized by Amino Allyl MessageAmp II aRNA Amplification Kit (Ambion#1753) using total RNA. CyeDye Coupling and fragmentation were performed, then hybridized for 16 h at 37℃ with rotary shake (250 rpm). 3D-Gene Scanner (Toray Industries Inc., Tokyo, Japan) was used for scanning. The signals were globally normalized and background subtracted. The raw data was deposited in GEO (Accession number: GSE126812).

### Real-time PCR

Total RNA was extracted from the cells using ISOGEN (Nippon Gene, Toyama, Japan) according to the manufacturer’s instructions. First-strand cDNA was synthesized from total RNA using a Superscript II RT kit (Invitrogen). Using this cDNA as a template, relative quantifications of various gene expression levels were conducted with real-time PCR. The primers and probe sets of target genes were designed with the manufacturer’s software from a Universal Probe Library (UPL; Roche, Basel, Switzerland) and are listed (Table 1). *GAPDH* was used as the internal control. PCR analysis was performed using ViiA 7 ™ (Applied Biosystems, Foster City, CA, USA).

**Table 1.** Primer Sequences and Probe Number for Real-time PCR.

Target human genes	Foward primer	Reverse primer	Probe number
*POU5F1*	cttcgcaagccctcatttc	gagaaggcgaaatccgaag	60
*LIN28A*	ccgtgtccaaccagcagt	acgttgaaccacttacagatgc	83
*HNF4A*	cagcactcgaaggtcaagcta	acgggggaggtgatctgt	66
*AFP*	atggccatcaccagaaaaat	cataagtgtccgataataatgtcagc	66
*ALB*	gtgaggttgctcatcggttt	gagcaaaggcaatcaacacc	7
*CYP3A4*	gatggctctcatcccagactt	agtccatgtgaatgggttcc	2
*ACTN2*	catgatccaggaggaggaggagt	acaccaggcagtgaaggtct	7
*NKX2.5*	cacctcaacagctccctgac	aatgcaaaatccaggggact	7
*GAPDH*	agccacatcgctcagacac	gcccaatacgaccaaatcc	60

### Immunohistochemistry

The cells were fixed with 4% paraformaldehyde in phosphate-buffered saline (pH 7.0) for 5 min. To stain nuclear proteins, the cells were permeabilized with 0.2% Triton X-100 (Sigma) for 5 min. Subsequently, the cells were treated with ImmunoBlock^®^ (DS Pharma Biomedical, Osaka, Japan) for 30 min at 25℃. The cells were treated with primary antibody in a 1:1 solution of ImmunoBlock^®^ and TBS containing 0.1% Tween-20 overnight at 4℃. Secondary antibody treatment was conducted for 30 min at 25℃. After nuclear staining with DAPI, fluorescence signals were observed via fluorescence microscopy (IX71; Olympus, Tokyo, Japan). Alternatively, fluorescence signals of the cell populations were analyzed via FACS. The primary and secondary antibodies are listed (Table 2).

**Table 2.** Antibody List for Immunocytochemistry.

**Target Antigen**	**Primary antibodies**	**Manufacturer**	**Catalog No.**
Alcam/CD166	Goat Polyclonal IgG	R&D	AF1172
HNF4α	Rabbit Polyclonal IgG	Santa cruz	SC-8987
α-Fetoprotein (AFP)	Mouse monoclonal IgG2a (clone C3)	Sigma-Aldrich	A8452
Albumin	Rabbit Polyclonal IgG	Dako	A0001
Human Nuclei	Mouse monoclonal IgG1 (clone 235-1)	Chemicon	MAB1281
α-Actinin (Sarcomeric)	Mouse monoclonal IgG1 (clone EA-53)	Sigma-Aldrich	A7811
Oct-3/4	Mouse monoclonal IgG1	BD	611202
**Target Species**	**Secondary antibodies**	**Manufacturer**	**Catalog No.**
Goat IgG (H+L)	Donkey Alexa Fluor 488	Invitrogen	A-11055
Goat IgG (H+L)	Donkey Alexa Fluor 546	Invitrogen	A-11056
Rabbit IgG (H+L)	Donkey Alexa Fluor 488	Invitrogen	A-21206
Rabbit IgG (H+L)	Donkey Alexa Fluor 546	Invitrogen	A-10040
Mouse IgG (H+L)	Donkey Alexa Fluor 488	Invitrogen	A-21202
Mouse IgG (H+L)	Donkey Alexa Fluor 546	Invitrogen	A-10036

### Statistical analysis

Real-time PCR data obtained from three independent FACS experiments were expressed as mean ± standard deviation (SD). Statistical analyses were performed using EZR software (Jichi Medical University, Japan) ^(17)^. For multiple comparisons, significant differences were determined by one-way analysis of variance (one-way ANOVA) followed by post-hoc testing with the Tukey–Kramer test. For two sample comparisons, Student’s *t*-test was performed. Statistical significance was set at p < 0.05.

## Results

### Purification of spontaneously differentiated hepatocyte-like cells of human ESC

**Figure 1.**
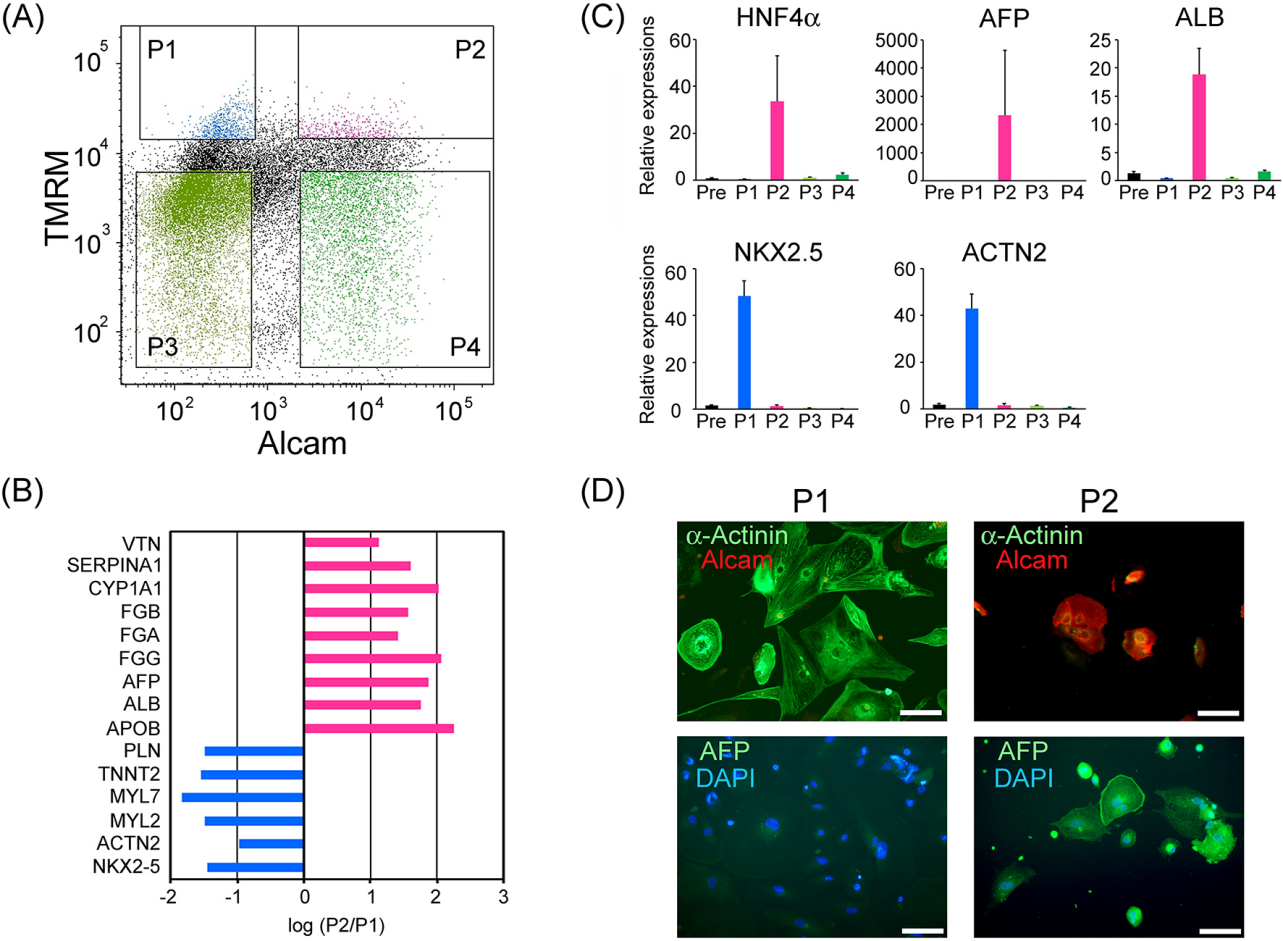
Separation of hepatocyte-like cells and cardiomyocytes from human ESC-derived EBs. (A) FACS separation of human ESC-derived EB cells by TMRM and ALCAM labeling with the antibody. (B) Signal differences between P1 and P2 in representative hepatic and cardiac gene expression in mRNA microarray analysis. (C) Confirmation of hepatic and cardiac genes by quantitative real-time PCR in presorted-cells (Pre) and sorted cells (P1, P2, P3, and P4). All data are represented as mean ± standard deviation (SD) (n = 3). Statistical analyses were conducted via one-way ANOVA and Tukey–Kramer test. *: p < 0.05. (D) Immunohistochemical detection of α-actinin, ALCAM, and AFP in cultured P1 and P2 cells. Scale bars represent 100 μm.

To discover new cell surface markers combining with TMRM for the purification of cardiomyocyte sub-populations, we performed screening experiments for the antibody-library. We unexpectedly observed that an antibody for ALCAM segregated with a characteristic cell type whose features were clearly different from those of cardiomyocytes. We found that immunocytochemical staining for ALCAM could separate a high mitochondria-containing population into two (Figure 1A). Among the two populations with high TMRM signal, ALCAM-negative (-) cell population (P1) and ALCAM-positive (+) cell population (P2) were sorted separately. Total RNA of each group was extracted and applied to the comparative mRNA microarray analysis (Toray Industries, Inc., Tokyo, Japan; Chip name: Human 25k ver2.10). The results are summarized (Figure 1B), suggesting that P1 showed markedly higher expression levels of cardiomyocyte-specific genes (*PLN*, *TNNT2*, *MYL7*, *MYL2*, *ACTN2*, and *NKX2.5*), whereas, by contrast, P2 showed markedly higher levels of hepatocyte characteristic genes (*VTN*, *SERPINA1*, *CYP1A1*, *FGB*, *FGA*, *FGG*, *AFP*, *ALB*, and *APOB*). To verify the above observations, real-time PCR analyses were performed for hepatic and cardiac marker genes in all four populations and presort-cells (Pre). As a result, P2 showed markedly high mRNA expression of hepatocyte-characteristic genes (*HNF4A*, *AFP*, and *ALB*) and low cardiomyocyte-specific genes (*NKX2.5*, *ACTN2*). On the other hand, P1 showed opposite cell-type genes than P2. P3 and P4 with low TMRM signals showed minimal expressions of both of hepatocyte- and cardiomyocyte-related genes. Immunostaining for cardiomyocytes (α-actinin) and fetal hepatocytes (α-fetoprotein: AFP) supported mRNA expression properties of these populations (Figure 1C, D). The above results strongly suggest that hepatocyte-like cells can be selected from human ESC-derived EBs using a combinatorial marker of high mitochondrial content and ALCAM-positive expression.

### Enrichment of hepatoblasts from fetal mice

**Figure 2.**
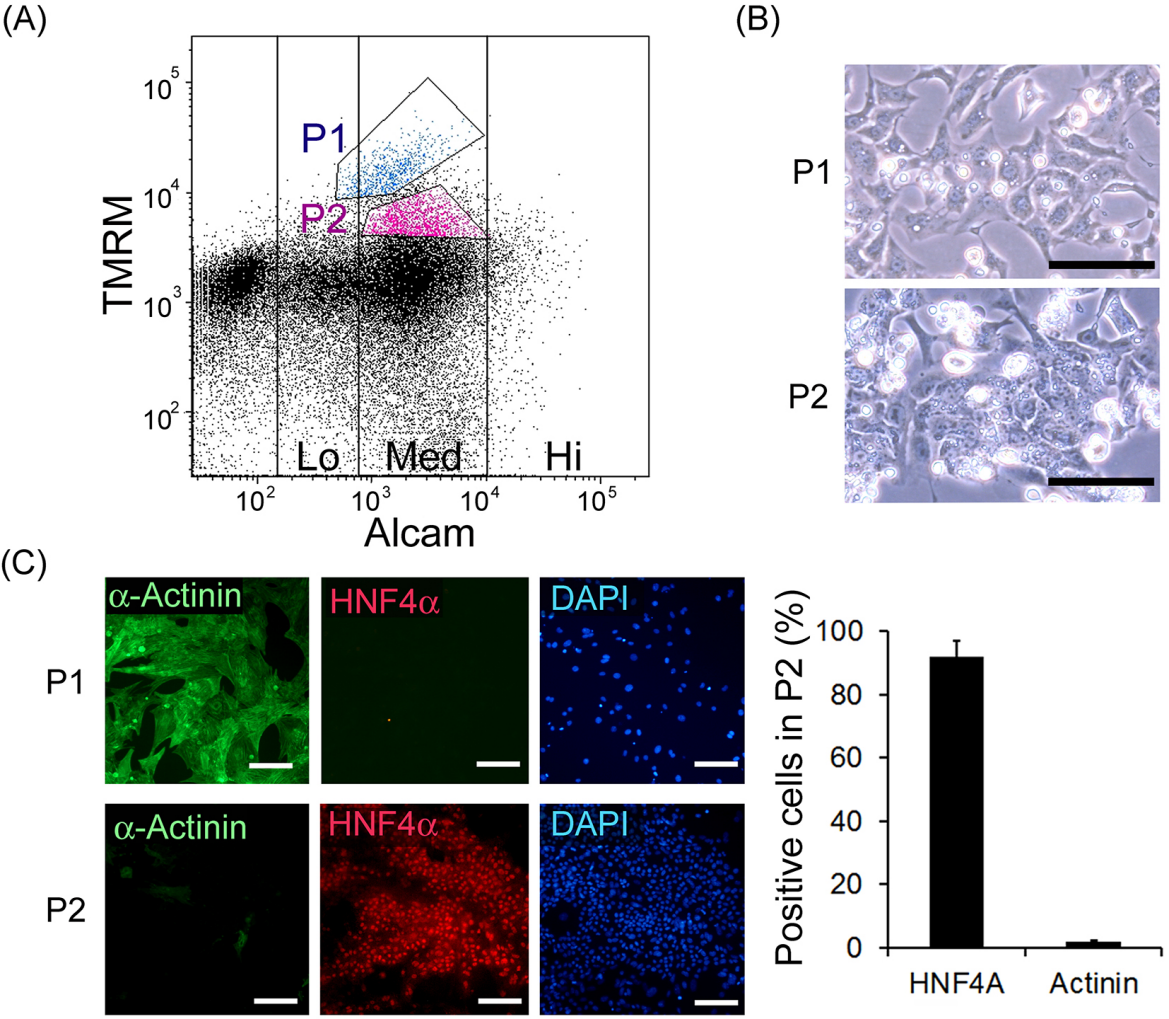
Enrichment of primary hepatocytes from mouse embryo. (A) FACS separation of total visceral cells of the embryonic day-14.5 fetuses based on TMRM and immunocytochemical ALCAM detection. (B) Phase contrast images of cultured P1 and P2 cells. Scale bars represent 100 μm. (C) Immunohistochemical detection of α-actinin and HNF4α. Scale bars represent 100 μm. The fractions of HNF4α or α-actinin positive cells per total nucleus were evaluated from each of four randomly selected areas.

In order to investigate the applicability of this method for mouse fetal liver cells, the whole viscera of embryonic day 14.5 mouse were applied to FACS analysis and living mitochondrial (TMRM) and immunological ALCAM staining was performed (Figure 2A). According to previous reports ^(13), (18)^, population 1 (P1), which showed the highest TMRM signal and ALCAM (Med), and population 2 (P2), which showed the second highest TMRM signal and ALCAM (Med), were sorted separately. These sorted cells were cultured on Matrigel-coated dishes for 5 d. These cells displayed typical morphologies of cardiomyocytes and hepatoblasts, respectively (Figure 2B). Immunohistochemistry confirmed that P1 cells were cardiomyocytes based on their α-actinin positivity, while P2 cells were confirmed to be hepatic cells based on their HNF4α positivity (Figure 2C). P2 contained 91.7 ± 5.3% of the HNF4α positive cells and 2.1 ± 0.4% of α-actinin positive cells, indicating that it might not seriously contaminate other organs like kidney or intestinal cells. It is noteworthy that mouse fetal cardiomyocytes expressed ALCAM, while the human pluripotent stem cell-derived cardiomyocytes did not (shown below). Furthermore, to investigate the relationship to previously reported methodology, we performed FACS analysis for Dlk1 in addition to TMRM and ALCAM and found P2 cells were also positive for Dlk1 (Supplementary Figure 1).

### Evaluation of modified directed differentiation method for definitive hepatocyte-like cells from human iPSCs

**Figure 3.**
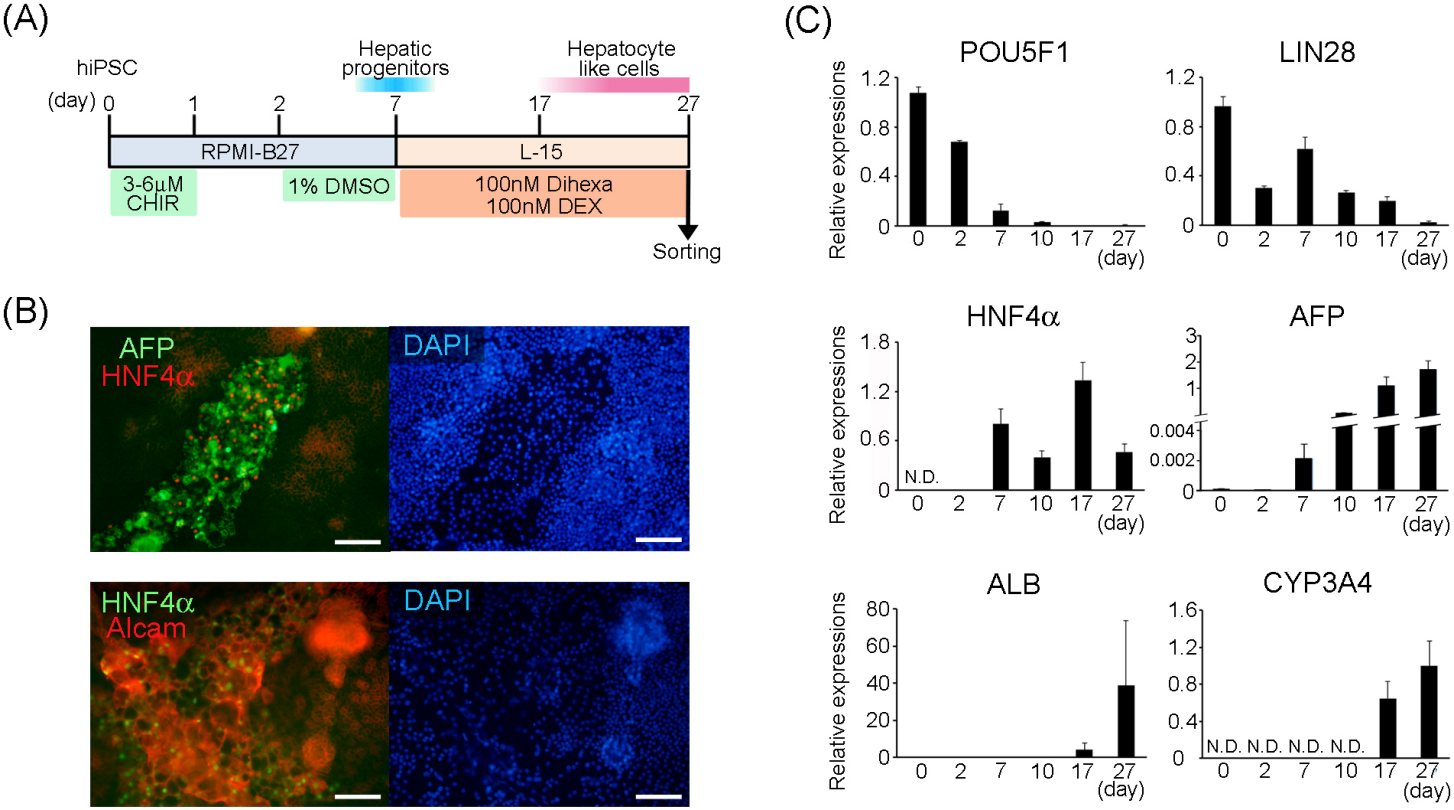
Hepatocyte-directed differentiation of human iPSCs. (A) Timeline of hepatocyte directed differentiation. (B) Quantitative analysis of pluripotent and hepatic related gene expression by real-time PCR. All data are represented as mean ± standard deviation (SD) (n = 3). N.D. shows “not detected”. (C) Immunohistochemical detection of AFP, HNF4α, and ALCAM on day 27 of differentiation culture. Scale bars represent 200 μm.

We basically followed the previously-reported efficient hepatocyte differentiation method ^(5)^, to induce definitive differentiation of hepatocyte-like cells. We modified the method for re-seeding of hepatic progenitor cells onto an a Matrigel-coated cell dish and culturing them for up to 20 d in the maturation medium. Along with the differentiation schedule (Figure 3A), mRNA expression including the undifferentiated (*POU5F1*, *LIN28*), hepatic progenitor (*HNF4A*, *AFP*), and hepatocyte maturation (*ALB*, *CYP3A4*) marker genes was analyzed by quantitative real-time PCR on days 0 (undifferentiated iPSCs), 2 and 7 (termination of induction of hepatic progenitor cells by dimethyl sulfoxide) and days 10, 17, and 27 (early, middle, and late period of definitive hepatocyte-differentiation, respectively); (Figure 3B). The expression levels of stem cell-marker genes: *POU5F1* and *LIN28* progressively decreased with the progression of differentiation. By contrast, differentiation marker genes were upregulated. *HNF4A* and *AFP* expressions increased rapidly from day 7. Expression of the former continued thereafter, but that of the latter continued to increase. *ALB* expression was increased approximately 100-fold between days 10 and 17 and further increased by approximately 9.4-fold between days 17 and 27. Additionally, the expression of *CYP3A4*, a representative drug-metabolizing enzyme, was detected from day 17, and its expression increased further by day 27. These results suggested that our methodological modifications successfully promoted the induction of definitive hepatocyte differentiation. Immunohistochemical analysis of the differentiating cells on day 10 indicated that HNF4α-positive cells were expressing both of AFP and ALCAM proteins.

### Purification of human iPSC-derived hepatocyte-like cells using the combinatorial marker

**Figure 4.**
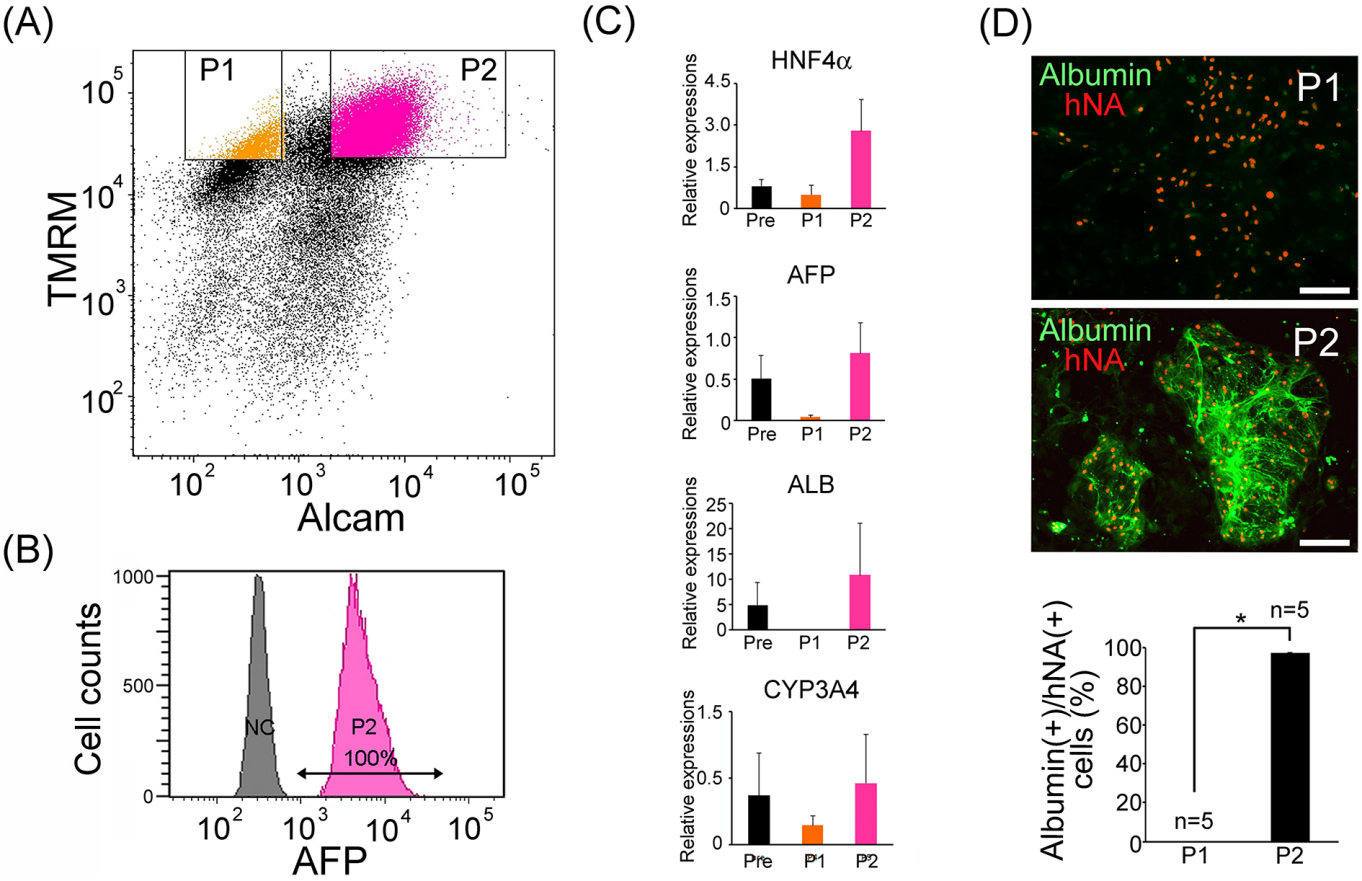
Purification of human iPSC-derived hepatocyte-like cells from the directed differentiation culture. (A) FACS based separation of hepatocyte directed differentiated cells derived from human iPSCs by TMRM and cell surface ALCAM labeling with the antibody. (B) Direct FACS analysis of AFP expression in sorted P2 cells (NC: negative control: without first antibody). (C) Quantitative analysis of hepatic related gene expression using real-time PCR in presort (Pre)-, P1, and P2 cells. All data are represented as mean ± standard deviation (SD) (n = 3). Statistical analyses were conducted via one-way ANOVA and Tukey–Kramer test. * = p < 0.05. (D) Immunohistochemical detection of albumin and human nuclear antigen (hNA) in cultured P1 and P2 cells (scale bars represent 100 μm), and quantification of albumin (+) cell-fraction in hNA (+) cells. Data are represented as mean ± standard deviation (SD) (n = 5). Statistical analysis was conducted via student’s *t*-test. * = P < 0.05.

FACS analysis using the two parameters was applied to cells from the hepatocyte-directed differentiation on day 27. The results suggested that the directed differentiation method increased possible hepatocyte-like cell population (P2) from 0.8 ± 0.4% (n = 3) of spontaneous differentiation to 32.3 ± 9.04% (n = 3); (Figure 4A). Sorted P2 cells were re-analyzed with immunocytochemical staining for AFP by FACS. As a result, all P2 cells were AFP-positive (Figure 4B). To confirm separation efficacy, relative gene expression levels of hepatocyte-specific genes (*HNF4A*, *AFP*, *ALB*, *CYP3A4*) in presorted, P1 and P2, cells were evaluated via real-time PCR. P2 cells were confirmed as hepatocyte-like cells with high expression levels of hepatocyte-specific genes (Figure 4C). Sorted P1 and P2 were cultured on MEFs. P2 cells tightly bound to each other and appeared as round colonies, while P1 cells were dispersed and showed a flatter morphology. Immunohistochemical detection of human nuclear antigen (hNA) and albumin confirmed the enrichment of hepatocyte-like cells in P2 (Figure 4D). To evaluate hepatocyte purities in P1 and P2 cells, the proportion of albumin-positive cell number/hNA-positive cell number was counted (n=5). It was found that 0% of P1 cells and 97 ± 0.38% in P2 cells were human iPSC-derived hepatocyte-like cells (Figure 4D). Interestingly, most AFP and albumin double-positive hepatocyte-like cells were collected in the TMRM (hi) and ALCAM (+) populations (Figure 4B and D). Furthermore, in order to investigate the relationship to reported methodologies, we performed FACS analysis for CD13 and CD133 or ASGR1 in addition to TMRM and ALCAM. It was found that P2 cells contain most of all CD13 and CD133 double positive cells and ASGR1 positive cells (Supplementary Figure 2 and 3, respectively). Next, to search the possibility of undifferentiated pluripotent stem cell-contaminations, we performed real-time PCR analysis for oct3/4, which suggested that oct-3/4 mRNA expression level in P2 was markedly lower than presort cells, and similar to fetal liver cells (Supplementary Figure 4A). To evaluate the safety of purified human iPSC-derived hepatocyte-like cells, we performed a colony forming assay of 50,000 P2 cells in 3.5 cm culture dish via long-term cultivation with stem cell culture medium containing mouse embryonic fibroblasts, and confirmed that they did not develop any stem cell colonies other than hepatocyte-like cell colonies (Supplementary Figure 4B).

## Discussion

As previously reported by Asahina ^(13)^, we observed that fetal mouse hepatoblasts expressed medium ALCAM levels. In addition, we confirmed that the second highest mitochondrial membrane potential-derived fluorescent signal was another effective separation marker. The purification method for human hepatocyte-like cells reported here may enrich almost all albumin- and HNF4α-expressing cells. This is an important improvement over the ASGR1-based method, which segregated an unknown sub-population from HNF4α and albumin expressing hepatocyte-like cells ^(12)^. Furthermore, in our study, ASGR1 positive cells were 17.6% of total cells, whereas our gating cells were 32.3%. Furthermore, our gating population contained largely CD13 and CD133 double positive populations (5.3% in total cells), suggesting that our gating strategy may collect a wider variety of hepatocyte-like cells.

ALCAM was reported as a marker for human hepatic stellate cells differentiated from iPS cells via mesoderm ^(14)^. This study indicated that ALCAM is expressed in definitively differentiated human hepatocyte-like cells expressing albumin ^(19)^. We suspect that the directed method of differentiating hepatocyte-like cells via the endoderm may not differentiate stellate cells. Furthermore, Hepatocyte-like cells might have higher amount of mitochondria than stellate cells as show here in FACS analysis using mouse whole viscera.

Several reports have indicated the engraftment and proliferation of transplanted primary hepatocytes on induced liver injury models ^(20), (21)^. Hepatocyte transplantation may represent a possible alternative to organ transplantation. However, it is well-known that isolated primary hepatocytes rapidly lose differentiated functional phenotypes including expression of various detoxifying enzyme. To avoid this, 3D culture by cell aggregation ^(22)^, co-culture with other non-hepatic cells ^(23), (24)^, and organoid formation ^(25)^ have been attempted. Despite these trials, functionally mature phenotypes of hepatocytes cannot be stabilized *in vitro*. Recently, it was suggested that transplanted human iPSCs-derived “liver buds” may reach maturity in a mature level overwhelming the *in vitro* systems ^(26)^. The *in vivo* maturation of human pluripotent stem cell-derived liver bud is a hopeful strategy for future human regenerative therapy. As reported, Catherine et al. found that possible tumor formation after transplantation may be an inevitable risk in 95% of pure human ES cell-derived hepatocytes ^(6)^. To our knowledge, negation of tumorigenicity in long-term transplantation of unpurified human pluripotent stem cell-derived hepatocytes has not yet been achieved. Our hepatocyte purification method may contribute to the production of safer hepatocytes suitable for liver regenerative therapies, although further studies are required for the safety verification before application in regenerative therapies.

In the development of pharmaceuticals, drug candidates should be checked with regard to liver metabolism and toxicity using human hepatocytes. However, human hepatocytes cannot be easily obtained because of ethical reasons. Practically, tumor hepatic cell lines like HepG2 are applied to daily screenings. However, they have very low expression of some important metabolic enzymes, such as cytochrome P450 ^(26)^. On the other hand, human iPSCs can be infinite sources of normal human hepatocytes cells. It is believed that purified human iPSC-derived hepatocyte-like cells will contribute to human hepatocyte assay systems.

## Article Information

### Conflicts of Interest

None

### Sources of Funding

This work was supported by the Ministry of Education, Culture, Sports, Science and Technology grant number [23390072].

### Author Contributions

All of the authors meet the four criteria.

### Approval by Institutional Review Board (IRB)

The experimental procedure and the protocol were approved as the application No.14045 by the Animal Care and Use Committees of the Keio University, Japan.

## Supplement

Supplementary Material
