# Supplementary material for: Hepatocyte-like Cells Derived from Human Pluripotent Stem Cells Can Be Enriched by a Combination of Mitochondrial Content and Activated Leukocyte Cell Adhesion Molecule
Source: JMA J. 2019 Aug 6;2(2):174–83. doi: 10.31662/jmaj.2018-0042 (PMC7889733; doi:10.31662/jmaj.2018-0042)
Supplement: Supplementary file 1 — Supplementary Material [file 2433-3298-2-2-0174-s001.pdf]

Supplementary Materials

Supplementary Figure 1

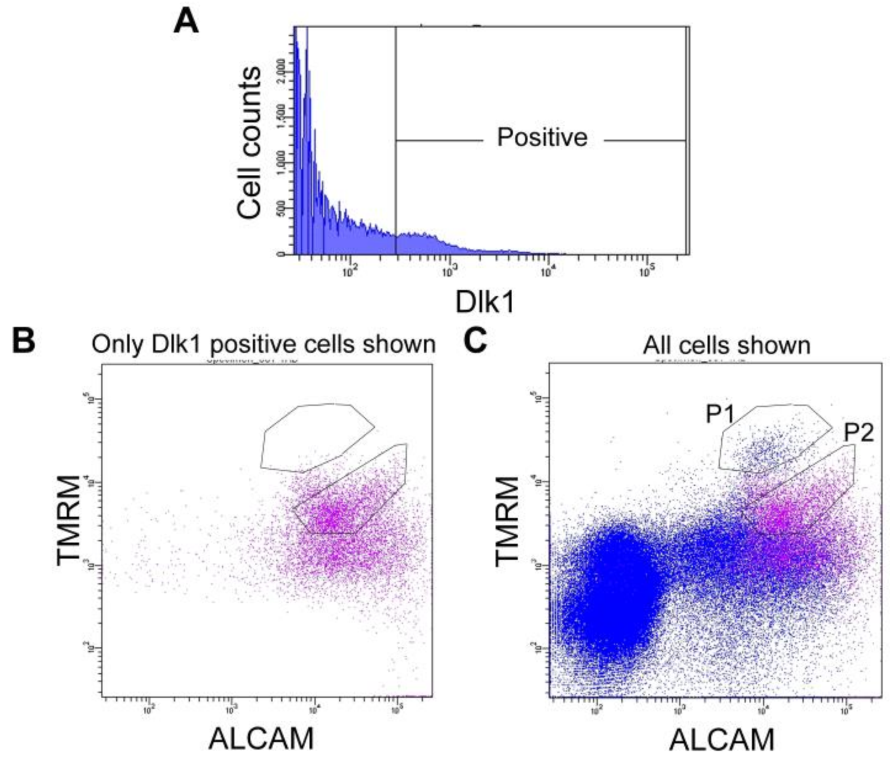

Supplementary Figure 1. Relationship TMRM(med) and ALCAM(med) to Dlk1 positive populations in mouse embryo. (A) FACS separation of total visceral cells of embryonic day-14.5 fetuses based on Dlk1. (B) Dlk1 positive cells were separated on TMRM and ALCAM. (C) Dlk1 positive cells in all cells separated on TMRM and ALCAM.

Supplementary Figure 2

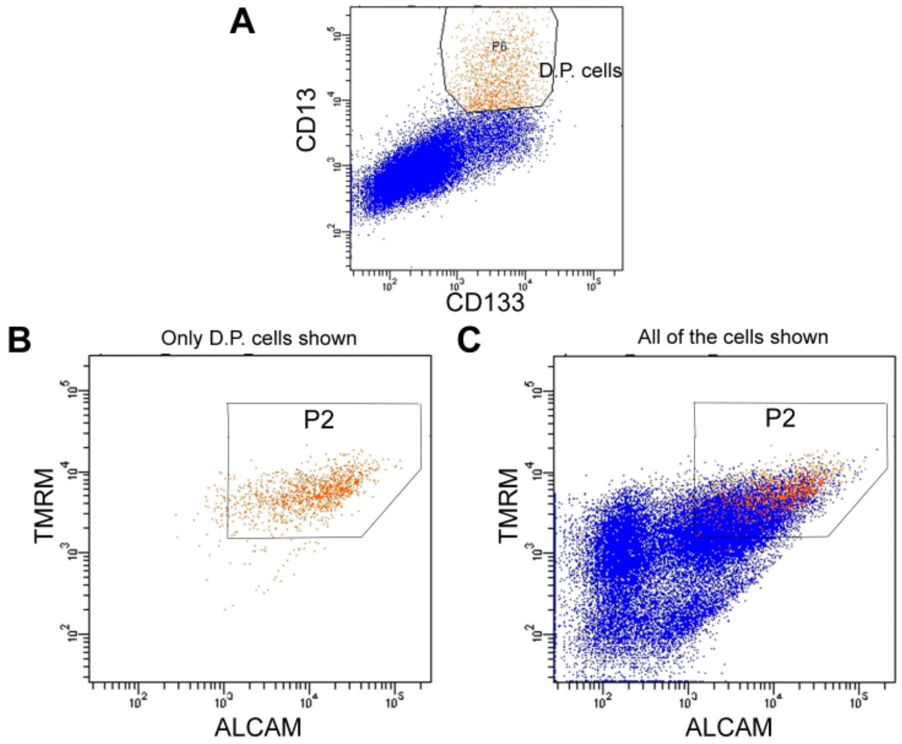

Supplementary Figure 2. Relationship of TMRM (hi) and ALCAM (hi) to CD13 and CD133 double positive populations in hepatocyte directed differentiated cells derived from human. (A) FACS separation of day-27 hepatocyte directed differentiated cells by CD13 and CD133. (B) CD13 and CD133 double positive cells were separated on TMRM and ALCAM. (C) CD13 and CD133 double positive cells in all cells based on TMRM and ALCAM.

Supplementary Figure 3

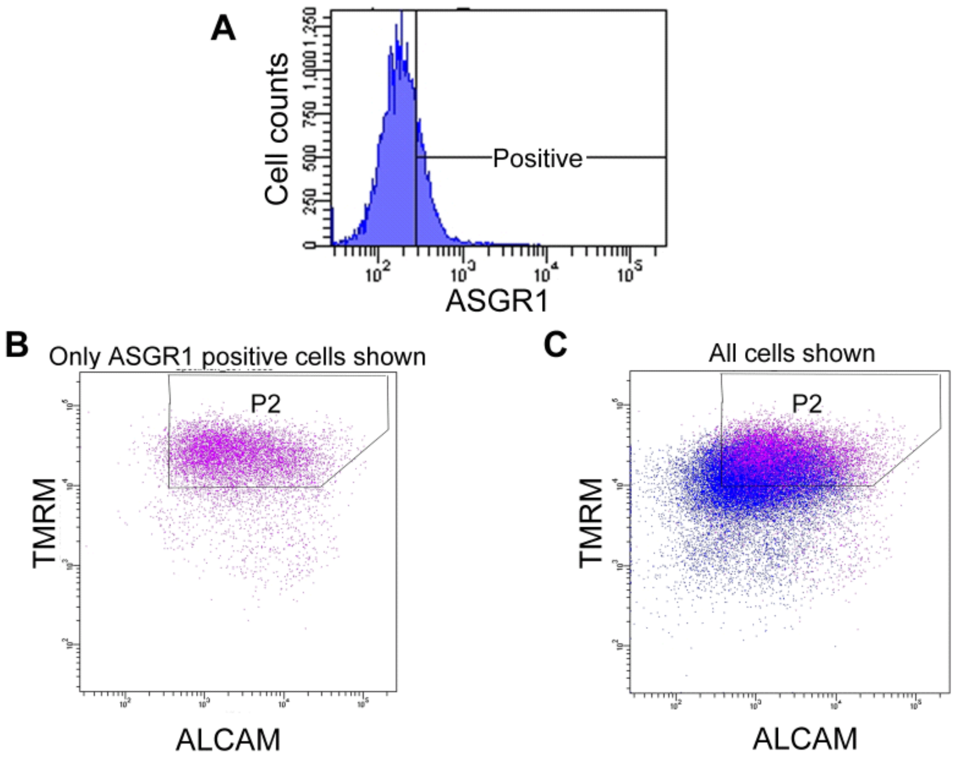

Supplementary Figure 3. Relationship of TMRM (hi) and ALCAM(hi) to ASGR1 positive populations in hepatocyte directed differentiated cells derived from humans. (A) FACS separation of day-27 hepatocyte directed differentiated cells by ASGR1. (B) ASGR1 positive cells were separated on TMRM and ALCAM. (C) ASGR1 positive cells in all cells based on TMRM and ALCAM.

Supplementary Figure 4

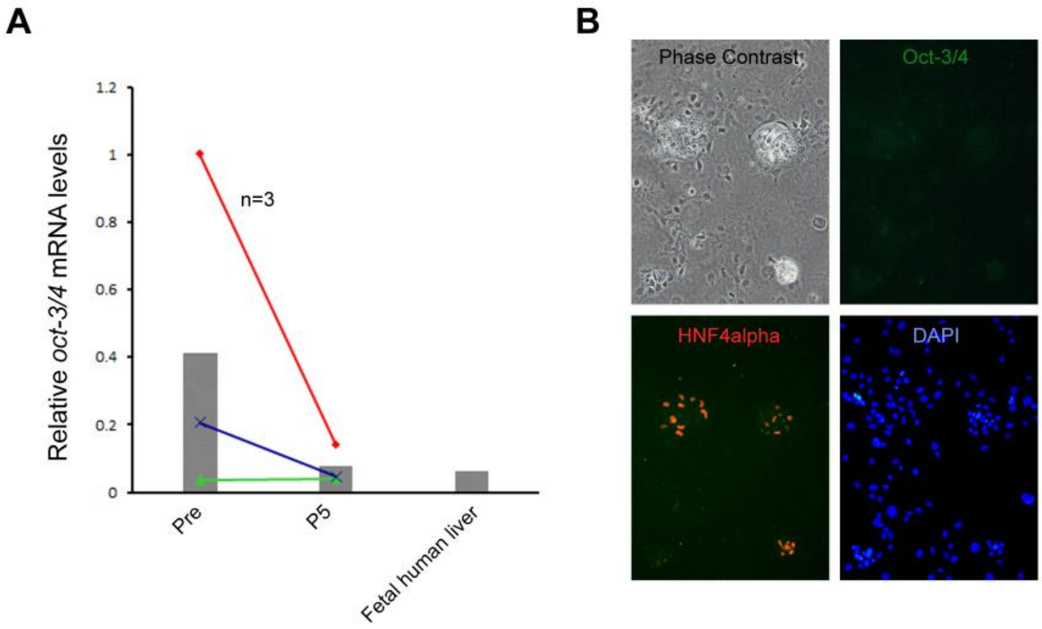

Supplementary Figure 4. Confirmation of purified TMRM (hi) and ALCAM (hi) population from hepatocyte directed differentiated cells derived from humans. (A) Quantitative analysis of hepatic related gene expressions by real-time PCR in presort (Pre), P2, and human fetal liver cells. Pre and P2 were performed in three independent samples. mRNA of human fetal liver was purchased from Clonetech. (B) Immunohistochemical staining regarding Oct-3/4 and HNF4 α on the P2 cells under stem cell culture condition with mouse embryonic fibroblasts for 14 days.
